# Oxidative Dissolution Process of Sphalerite in Fe_2_(SO_4_)_3_-O_3_ System: Implications for Heavy Metals Removal and Recovery

**DOI:** 10.3390/toxics12040275

**Published:** 2024-04-08

**Authors:** Mingtong Zhang, Hongbo Zhao, Yisheng Zhang, Xin Lv, Luyuan Zhang, Li Shen, Liang Hu, Jiankang Wen, Louyan Shen, Xianping Luo

**Affiliations:** 1 School of Minerals Processing & Bioengineering, Central South University, Changsha 410083, China; zmtincsu@csu.edu.cn (M.Z.); zys666@csu.edu.cn (Y.Z.); bqt2100301002@student.cumtb.edu.cn (X.L.); zhangluyuan@csu.edu.cn (L.Z.); lishen@csu.edu.cn (L.S.); huliang2018@csu.edu.cn (L.H.); 2 National Engineering Research Center for Environment-Friendly Metallurgy in Producing Premium Non-Ferrous Metals, GRINM Group Co., Ltd., Beijing 100088, China; 3 China Nerin Engineering Co., Ltd., Nanchang 330103, China; shenlouyan@nerin.com; 4 College of Resources and Environment, Jiangxi University of Science and Technology, Ganzhou 341000, China; luoxianping9491@163.com

**Keywords:** heavy metals, leaching, dissolution process, metal sulfides, sphalerite

## Abstract

Metal sulfides in waste rocks and tailings are susceptible to serious soil and water contamination due to the generation of acid mine drainage (AMD) during stockpiling. The hydrometallurgical process is one of the most essential heavy metal remediation technologies through harmless disposal and resource utilization of the waste sulfides. However, atmospheric hydrometallurgy of sulfides still faces great challenges due to low leaching efficiency and high cost. In this work, we proposed a cooperative leaching system (Fe_2_(SO_4_)_3_-O_3_) and investigated the oxidative dissolution process of sphalerite (ZnS). Under the optimal conditions, the extracted zinc reached 97.8%. Reactive oxygen species (ROS) (·OH, ^1^O_2_ and ·O_2_^−^) were identified in the radical quenching experiments. The dissolution of sphalerite did not show passivation due to the ozone’s capability to oxidize the sulfur in sphalerite to sulfate. In addition, stirring rate, O_3_ inlet concentration, and Fe_2_(SO_4_)_3_ concentration had a significant effect on the dissolution of sphalerite. Meanwhile, the apparent activation energy was 24.11 kJ/mol based on kinetic fitting, which indicated that the controlling step of the reaction was mainly a diffusion process. This work demonstrated the cooperative effect of sphalerite leaching in the O_3_-Fe_2_(SO_4_)_3_ system and provided a theoretical reference for efficient and atmospheric dissolution of sphalerite.

## 1. Introduction

Currently, froth flotation is the most widely used method of separating sulfide ores to obtain a sulfide concentrate for smelting [1]. The waste ore after flotation is called flotation tailings, containing large amounts of heavy metal ions and sulfides. As a result, in recent decades, this method has produced billions of tons of flotation tailings along with the operation of mining plants around the world [2]. Moreover, the mining process also produces large quantities of sulphureous mining waste every year. These sulfide metals mainly include pyrite, sphalerite, chalcopyrite, and galena [3]. As sulphureous waste ore is oxidized during stockpiling, the acid mine drainage (AMD) produced could lead to serious soil and water pollution through the ground or surface water cycle.

Tailing dam and surface paste methods have been developed for more economical and safe storage of tailing [4]. However, with the increasing number of tailings storage depots, tailing management has become more difficult and the risk of harm to the environment has increased. In addition, there are still a considerable number of valuable metals in these tailings that are worth extracting and utilizing. In recent years, a number of new methods are being investigated to recover valuable metal components and to reduce the hazards of sulphureous waste ore [5,6]. Hydrometallurgy is often used to dissolve low-grade minerals of complex composition. Meanwhile, the process is one of the most essential heavy metal remediation technologies through harmless disposal (heavy metals removal) and resource utilization (heavy metals recovery) of the waste sulfides.

The bioleaching process has been used in experimental and industrial-scale studies [7,8] due to low cost, large production scale and low mineral grade requirements. *Acidithiobacillus ferrooxidans* is commonly investigated and used microorganisms for leaching sulfide ores [9]. In the presence of this bacteria, pyrite (FeS_2_), the prime component of the sulfide tailings, oxidized to trivalent iron and sulphate [4]. Moreover, many studies have shown that zinc, iron, and lead sulfide undergo bioleaching to produce the relevant sulfates and elemental sulfur [3]. However, elemental sulfur can lead to the results of slow reaction rate and inadequate reaction [10,11,12], because of the formation of the passivation layer. The sulfur layer is most likely formed when the sulfur in sulfide ores is oxidized by trivalent iron. Santos, Rivera-Santillán, Talavera-Ortega, and Bautista [13] studied sphalerite leaching at a pH of 1.0 and 0.5 M Fe^3+^ at 70 °C, using 44–53 μm particle sizes, and a Zn extraction of 70% after 7 h was obtained, and scanning electron microscopy images showed the presence of a passive layer on the surface of the undissolved sphalerite. Likewise, Nikkhou, Xia, and Deditius [12] dissolved sphalerite particles < 22 μm with 2.06 M Fe_2_(SO_4_)_3_ in a 1 M citric acid solution between 35–130 °C, and achieved Zn extraction < 80%. Furthermore, the sulfur layer and hydrated ferrous sulfate were confirmed by surface analysis of the samples.

Powerful oxidation can be used to remove the passivation of the sulfur layer. For instance, Santos, Rivera-Santillán, Talavera-Ortega, and Bautista [13] dissolved 106–150 μm sphalerite grains using 2.06 M Fe(NO_3_)_3_ at 90 °C, and achieved complete extraction within 5 h. Nitrate plays a role in converting elemental sulfur to sulfate. Likewise, [13] leached pyrite by bioleaching (*Leptospirillum ferrooxidans* and *Acidithiobacillus thiooxidans*) and found that the passivation layer generated caused inhibition of dissolution. After ozone activation, the sulfur layer on the surface was destroyed and the total iron leaching rate increased from about 34% to about 50%. Compared to other oxidizers, ozone has significant advantages such as high efficiency, environmental friendliness, and ease of generation. At the same time, these characteristics are also very conducive to the realization of the resource utilization of sulfide tailings.

Ozone is a powerful and environmentally friendly oxidant, with a redox potential of 2.07 V (SHE) in acidic solutions, which is widely used in the treatment of wastewater as an advanced oxidant [13,14]. Not only does it eliminate the sulfur layer, but it also does not introduce impurities into the leaching solution. In addition, the leachate can be continuously used for the sulfide tailings dissolution, which reduces the volume of wastewater. In recent years, many researchers have studied the utility of ozone in hydrometallurgy and the treatment of mine wastewater. These include gold ore [15,16], silver-bearing pyrite [17], pyrite [13], stibnite [18], sphalerite [19], wastewater containing cyanide [20], and thiosalts [21], etc.

However, the reaction mechanism of sulfide tailings with ozone is very complex because tailings contain multiple metal sulfide ores and the reaction process involves gas–liquid and liquid–solid mass transfer. In addition, there are two reaction pathways for ozone: direct reaction via ozone molecules and indirect reaction by ·OH (E = 2.80 V), which is generated by its decomposition [22]. Therefore, the generation of sulfur layers can be avoided by whichever pathway predominates. Although in the majority of cases, hydroxyl radicals are generated under alkaline conditions [22], they can be generated under acidic conditions in the presence of some catalysts [23]. Example include copper sulfide [24], galena [25], ferrous ions [14,26], and trivalent ferric ions [23].

If ozone and sulfide tailings are chosen as reactants, it is difficult to obtain a clear reaction mechanism. Thus, one sulphide ore can be selected to react with ozone to analyze the mechanism step by step. In this study, sphalerite was selected for research. In addition, the effect of Fe^3+^ should be taken into account in the leaching process, since the oxidation of pyrite by ozone produces Fe^3+^ with oxidizing properties [13]. Furthermore, Fe^3+^ may affect the pathway of ozone oxidation of sulfide ores due to more difficult gas–solid mass transfer. The original reaction path for the sphalerite oxygen pressure leaching process is Equation (1), and with the addition of Fe^3+^ it is Equations (2) and (3). A significant increase in the leaching rate with the addition of Fe^3+^ can be attributed to the increase in the rate of mass transfer.

(1) $$ZnS+H_{2}SO_{4}+0.5O_{2}\to ZnSO_{4}+H_{2}O+\frac{1}{8}S_{8}$$

(2) $$ZnS+Fe_{2}{\left(SO_{4}\right)}_{3}\to ZnSO_{4}+2FeSO_{4}+\frac{1}{8}S_{8}$$

(3) $$2FeSO_{4}+H_{2}SO_{4}+0.5O_{2}\to Fe_{2}{\left(SO_{4}\right)}_{3}+H_{2}O$$

Overall, the dissolution of sphalerite was systematically investigated in this study. The impacts of different dissolving conditions on the extracted zinc were evaluated. The reactive oxygen species in the O_3_-Fe_2_(SO_4_)_3_ system were identified by quenching experiments. The reuse of leachate in O_3_-Fe_2_(SO_4_)_3_ system was also evaluated by cyclic tests. In addition, the properties of the residue surface and electrochemical properties were systematically studied. The reaction mechanism and dissolution kinetics in the sphalerite dissolution process were explored. This study provides a valuable reference for the leaching of other sulfide minerals from tailings.

## 2. Materials and Methods

### 2.1. Materials

The sphalerite samples were obtained from Changsha, Hunan Province. These samples were then crushed, ground, sieved, and used for leaching experiments with particle sizes less than 38 µm. Moreover, the XRF (X-Ray Fluorescence) analysis showed that these sphalerite samples contained 63.76 wt% Zn, 26.01 wt% S, and 1.53 wt% Fe. The XRD (X-Ray Diffraction) results were consistent with this result, indicating that these samples are almost pure ZnS.

Ozone gas was generated from oxygen in the air using an ozone generator (Xianglu Environmental Co., Ltd., Changsha, China; XLK-G20). Fe_2_(SO_4_)_3_, H_2_SO_4_, KOH, benzoquinone (p-BQ), L-histidine, and tert-butyl alcohol (TBA) were analytical grade and purchased from Sinopharm Chemical Reagent Beijing Co., Ltd., Beijing, China. Distilled water was used to prepare the leach solution and the relevant properties of the distilled water samples are shown in Table 1.

### 2.2. Dissolution Experiments

For less evaporation, all leaching experiments were conducted in a 500 mL beaker covered with a watch glass. The beaker was placed in a water bath with magnetic stirring to maintain the temperature and stirring rate. Ozone was injected into the solution through a venting stone to increase the gas–liquid interface area. The leachates were taken regularly for inductively coupled plasma optical emission spectroscopy (ICP–OES) (ICAP 7400, Thermo Fisher Scientific Co., Waltham, MA, USA). Deionized water was used to compensate for the evaporated loss. The leach residues were filtered for surface analysis and electrochemical studies.

The aim was to evaluate whether efficient and environmentally friendly leaching of sphalerite can be achieved by introducing ozone into acidic ferric sulfate solutions. Leaching experiments were carried out to determine the effect of the zinc dissolution rate by varying the stirring speed (from 300 to 750 r/min), sulfuric acid addition (from 10 to 40 g/L), temperature (from 30 to 70 °C), ozone inlet concentration (from 0 mg/L to 95 mg/L), and ferric ion concentration (from 0.2 to 0.8 mol/L). All leaching experiments were maintained with a leaching solution of 200 mL, a ventilation rate of 1.2 L/min, a slurry solid–liquid ratio of 2%, and a leaching time of 6 h. Moreover, the concentration of ferrous ions was measured by enzyme standardization using o-phenanthroline as the indicator, and that of ferric ions was calculated.

In order to explore the reaction mechanism and kinetics of sphalerite dissolved in O_3_ + Fe_2_(SO_4_)_3_ system, the residues oxidized by different oxidation factors were used for surface analysis electrochemical tests. In addition, comparative experiments were conducted concerning ozone and ferric ions, without ozone and without ferric ions under otherwise identical leaching conditions. Then, their leachate and leachate residue were analyzed accordingly. Moreover, to determine whether reactive oxygen species were generated, scavenger experiments with benzoquinone, L-histidine and tert-butyl alcohol as the scavenger were conducted. All experiments were run in duplicate.

### 2.3. Analysis of the Residue Surface

The residue samples were filtered, dried, and used for surface analysis, which were dissolved separately for two hours in Fe_2_(SO_4_)_3_, O_3_, and O_3_ + Fe_2_(SO_4_)_3_ system, respectively.

The surface images of the residues were analyzed by scanning electron microscopy [27]. The S of different valence states on the leaching residue surface was detected by X-ray photoelectron spectroscopy (XPS) (ESCALAB 250Xi). The tests were conducted using an Al K Alpha X-ray source in a standard lens model. Besides, pass energies of 100 eV and 0.1 eV/step were adopted in the constant analyzer. Thermo Avantage 5.957 was used to fit the XPS spectra. Before fitting, the spectra were calibrated with C 1 s at a binding energy of 284.8 eV, and background subtraction was performed using the Smart method. Then, the spectra were peak fitted using the Gauss-Lorentz line (SGL) function according to the binding energy of the specific sulfur form.

The mineral composition of the residues was detected by X-ray diffraction (XRD, Advance D8/Bruker, Billerica, MA, USA). The microstructure and material composition of the residue samples surface were investigated by scanning electron microscopy (JSM-6490LV/JEOL, Tokyo, Japan) and energy dispersive spectroscopy (EDS) (Nepture XM 4/EDAX, Pleasanton, CA, USA). The possible reaction products on the surface of the residues were characterized by Raman spectroscopy (Horiba Scientific LabRAM HR Evolution, Piscataway, NJ, USA). An exciting source at 632 nm (He Ne laser source) was used and energy level was 5 mW.

### 2.4. Electrochemical Tests

Carbon paste electrodes made from sphalerite ore powder were processed for 2 h in Fe_2_(SO_4_)_3_, O_3_, and O_3_ + Fe_2_(SO_4_)_3_ system, respectively.

Carbon paste electrodes are made using mineral powder, including residues and untreated sphalerite, homogeneously mixed and pressed with graphite powder and paraffin wax in a ratio of 7:2:1. A three-electrode system was built, and a carbon paste electrode, Ag/AgCl, and carbon rod played the role of the working electrode, reference electrode, and counter electrode, respectively. Furthermore, the reference electrode was connected to the glass cell through a Luggin capillary with saturated KCl solution. Moreover, the electrolyte was a 10 g/L sulfuric acid solution in all tests. An electrochemical workstation (CHI700E, CH Instruments, Inc., Bee Cave, TX, USA) was used to complete these tests including open-circuit potential-time (OCPT), electrochemical impedance spectroscopy (EIS), and potentiodynamic polarization (Tafel plot).

## 3. Results and Discussion

### 3.1. Comparision of Sphalerite Leaching in Various Systems

#### 3.1.1. Dissolution of Sphalerite

**Figure 1:**
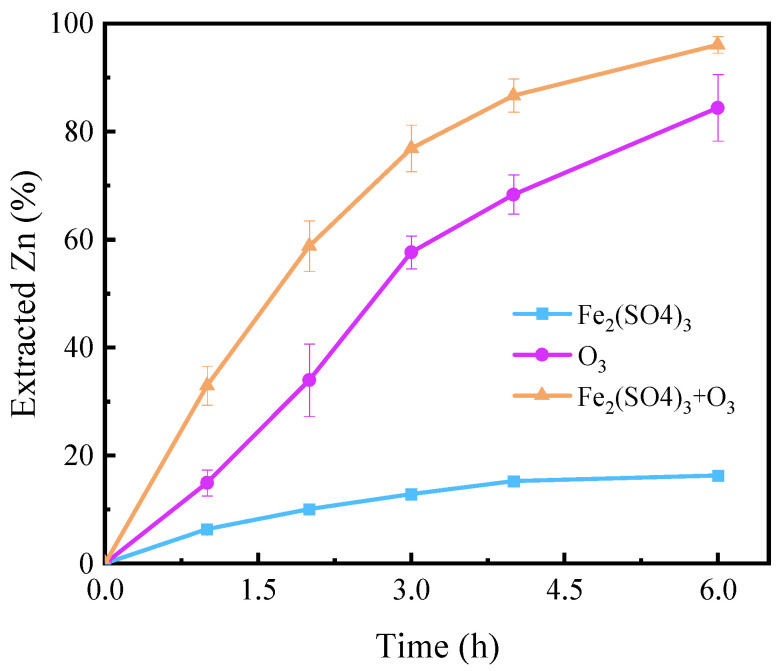
Dissolution of sphalerite in different systems. Experimental conditions: [Fe_2_(SO_4_)_3_] = 0.2 M, O_3_ inlet = 95 mg/L and 1.2 L/min, H_2_SO_4_ addition = 10 g/L, T = 40 °C, stirring rate = 650 rpm.

As shown in Figure 1, the extracted zinc in the Fe_2_(SO_4_)_3_, O_3_, and O_3_ + Fe_2_(SO_4_)_3_ systems was approximately 16.3%, 84.4%, and 97.8%, respectively. The distinction between these systems is whether or not they contain one or both of Fe_2_(SO_4_)_3_ and O_3_. Their concentrations and other experimental conditions were identical. The pH values of the leachate in the Fe_2_(SO_4_)_3_, O_3_, and O_3_ + Fe_2_(SO_4_)_3_ systems were 1.06, 0.94, and 1.06, respectively. H_2_SO_4_ addition was 10 g/L for all systems, but pH was higher in the presence of Fe_2_(SO_4_)_3_. This may be due to the complexation of SO_4_^2−^ with H^+^ reducing the free H^+^ concentration. Comparatively, the extracted Zn of O_3_ system was significantly higher than that of system Fe_2_(SO_4_)_3_. The combination of O_3_ and Fe_2_(SO_4_)_3_ had a substantial promoting effect on the dissolution of sphalerite. Especially in the first three hours, the extracted Zn of the O_3_ + Fe_2_(SO_4_)_3_ system is greater than the sum of that of O_3_ and Fe_2_(SO_4_)_3_ systems. On the one hand, we verified that the powerful oxidation effect of O_3_ can be used for sphalerite dissolution. On the other hand, we can speculate that there is a cooperative effect between O_3_ and Fe_2_(SO_4_)_3_. Likewise, O_3_ was used to enhance the dissolution of pyrite by oxidizing the passivation layer in a bioleaching system [13]. Furthermore, several studies indicated that Fe (II) and Fe (III) can promote the decolorization of Reactive Red 2 with O_3_ through catalysis [26,28]. It was proposed that more reactive substances are generated during the catalytic process, such as ·OH. Hence, the catalysis of O_3_ by Fe_2_(SO_4_)_3_ may be the reason why sphalerite dissolved faster in the O_3_ + Fe_2_(SO_4_)_3_ system. In addition, Fe_2_(SO_4_)_3_ plays a crucial role in enhancing oxygen mass transfer in pressure leaching of sphalerite [29]. Thus, it may also enhance O_3_ mass transfer in the dissolution of sphalerite in the O_3_ + Fe_2_(SO_4_)_3_ system.

#### 3.1.2. Role of Reactive Oxygen Species

Reactive oxygen species (ROS) (including ·OH, ^1^O_2_ and ·O_2_^−^) that are produced by the decomposition of ozone may play an essential role in dissolution of sphalerite in the O_3_ + Fe_2_(SO_4_)_3_ system [30]. In order to obtain insights into the mechanism of sphalerite dissolution, these typical ROS generated in the O_3_ + Fe_2_(SO_4_)_3_ system were identified by a radical quenching experiment. Normally, tert-butanol (TBA) is considered a sensitive scavenger for ·OH (k _(·OH, TBA)_ = 6.0 × 10^8^ M^−1^ S^−1^), and L-histidine is an excellent quencher of ·OH and ^1^O_2_ (k _(·OH, L-histidine)_ = 5.0 × 10^9^ M^−1^ S^−1^, k _(1O2, L-histidine)_ = 1.5 × 10^8^ M^−1^ S^−1^) [31]. In addition, para-benzoquinone (p-BQ) is typically selected as ·OH and ·O_2_^−^ scavenger (k _(·OH, p-BQ)_ = 4.5 × 10^9^ M^−1^ S^−1^, k _(·O2−, p-BQ)_ = (0.9 − 1.0) × 10^9^ M^−1^ S^−1^) [30]. Hence, TBA, L-histidine, and p-BQ were applied to identify ·OH, ·OH + ^1^O_2_, and ·OH + ·O_2_^−^.

**Figure 2:**
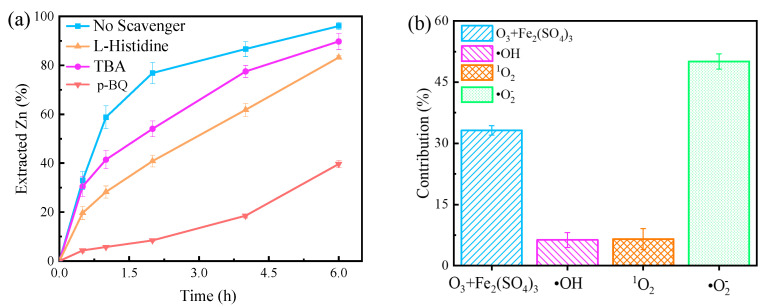
(**a**) Dissolution of sphalerite in the O_3_ + Fe_2_(SO_4_)_3_ system with different scavengers. (**b**) The contributions of O_3_ + Fe_2_(SO_4_)_3_ and ROS (·OH, ^1^O_2_, ·O_2_^−^) to the extracted Zn. Experimental conditions: [Fe_2_(SO_4_)_3_] = 0.2 M, O_3_ inlet = 95 mg/L and 1.2 L/min, H_2_SO_4_ addition = 10 g/L, T = 40 °C, stirring rate = 650 rpm; [L-histidine] = 0.1 M, [TBA] = 0.1 M, [p-BQ] = 0.1 M.

As illustrated in Figure 2a, the addition of 0.1 M TBA relatively inhibited sphalerite dissolution, especially within 1–3 h, which indicated that ·OH played a relatively vital role in sphalerite dissolution in the O_3_ + Fe_2_(SO_4_)_3_ system. Although in the majority of cases, ·OH is generated under alkaline conditions [22], it can be generated under acidic conditions in the presence of some catalysts (such as Fe (II) and Fe (III)) [23,26,28]. Furthermore, the addition of 0.1 M L-histidine resulted in a further inhibition of sphalerite dissolution, suggesting the presence of ^1^O_2_. The addition of 0.1 M p-BQ severely suppressed sphalerite dissolution, illustrating that ·O_2_^−^ is the dominant reactive oxygen species in the O_3_ + Fe_2_(SO_4_)_3_ system (Figure 2a). It was assumed that the quantity of ·OH generated and scavenged was the same in each set of experiments. Thus, it can be considered that the extracted Zn of “No scavenger” is contributed by O_3_ + Fe_2_(SO_4_)_3_ (direct effect), ·OH, ^1^O_2_ and ·O_2_^−^, “L-histidine” is contributed by O_3_ + Fe_2_(SO_4_)_3_ (direct effect), ^1^O_2_ and ·O_2_^−^, “TBA” is contributed by O_3_ + Fe_2_(SO_4_)_3_ (direct effect), and ·O_2_^−^, and “p-BQ” is contributed by O_3_ + Fe_2_(SO_4_)_3_ (direct effect). Calculations show that the contributions of O_3_ + Fe_2_(SO_4_)_3_ (direct effect), ·OH, ^1^O_2_, and ·O_2_^−^ are about 33.2%, 6.3%, 6.5%, and 50.1% in the O_3_ + Fe_2_(SO_4_)_3_ system, respectively (Figure 2b). However, it is noticeable that ROS scavenging experiments can be interfered with by many factors. Hence, the results are recommended for qualitative instead of quantitative evaluation.

#### 3.1.3. The Reuse of Leachate

**Figure 3:**
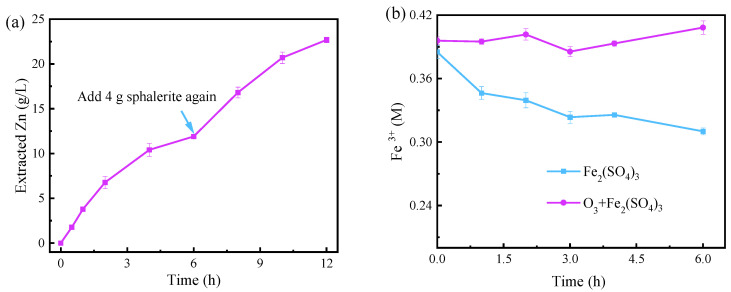
(**a**) Variation of the extracted Zn by reusing leachate to dissolve sphalerite in the O_3_ + Fe_2_(SO_4_)_3_ system. (**b**) Variation of Fe^3+^ concentration during sphalerite leaching in the O_3_ + Fe_2_(SO_4_)_3_ and Fe_2_(SO_4_)_3_ systems. Experimental conditions: [Fe_2_(SO_4_)_3_] = 0.2 M, O_3_ inlet = 95 mg/L and 1.2 L/min, H_2_SO_4_ addition = 10 g/L, T = 40 °C, stirring rate = 650 rpm.

To verify that the leachate can be reused in the O_3_ + Fe_2_(SO_4_)_3_ system, a sphalerite continuous dissolution experiment was conducted. As exhibited in Figure 3a, the Zn^2+^ concentration reached 11.9 g/L after 6 h dissolution, followed by the addition of minerals and continued dissolving for 6 h to reach 22.7 g/L. This result indicated that the solution after dissolving sphalerite in the O_3_ + Fe_2_(SO_4_)_3_ system had the potential to be reused several times. Furthermore, for understanding the mechanism, the concentration of Fe^3+^ was monitored during sphalerite dissolution in the O_3_ + Fe_2_(SO_4_)_3_ and Fe_2_(SO_4_)_3_ systems. As displayed in Figure 3b, the [Fe^3+^] was always maintained around the initial concentration (0.4 M) in the O_3_ + Fe_2_(SO_4_)_3_ system, while in the Fe_2_(SO_4_)_3_ system the [Fe^3+^] decreased continuously from the initial concentration. This equilibrium may be explained by the fact that ozone constantly produced Fe^3+^ from Fe^2+^. Hence, this system can be easily maintained in a state where sphalerite can be dissolved efficiently. Additionally, in order to further purify valuable metals, separation and wastewater treatment are usually required after the leaching process. The process that allows continuous leaching of minerals not only improves the efficiency of dissolution, but also reduces the cost of separation and wastewater treatment. Therefore, dissolving sphalerite or other sulfide ores in the O_3_ + Fe_2_(SO_4_)_3_ system is significant for the entire process of metal extraction.

### 3.2. The Effect of Different Dissolving Conditions on the Extracted Zinc

**Figure 4:**
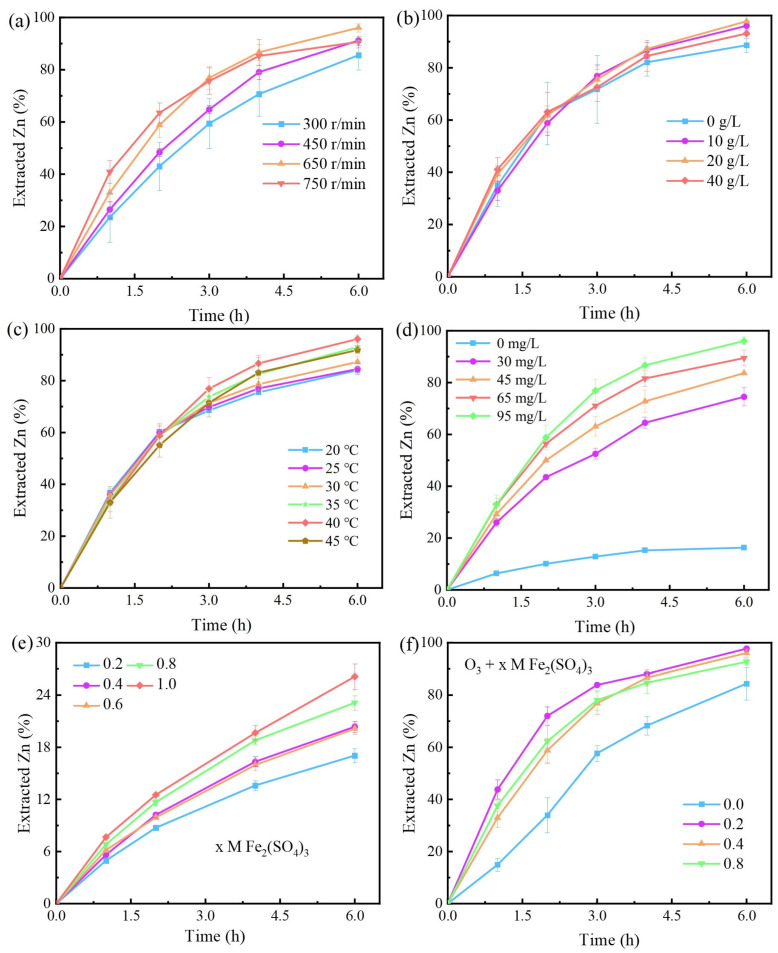
The effect of different factors on the extracted zinc: (**a**) stirring rate (rpm); (**b**) sulfuric acid addition; (**c**) temperature; (**d**) ozone inlet concentration; (**e**) Fe_2_(SO_4_)_3_ concentration, no ozone inlet; (**f**) Fe_2_(SO_4_)_3_ with 95 mg/L and 1.2 L/min O_3_ inlet.

The effect of stirring rate on the extracted zinc was evaluated, as illustrated in Figure 4a. The extraction of zinc increases with increasing stirring rate between 300 and 650 rpm. This trend can be attributed to the enhancement of O_3_ mass transfer in the leach solution [15], since the renewal rate of the gas–liquid contact surface increases with the stirring rate. In addition, the extracted zinc at 750 rpm was higher than that at 650 rpm during the first two hours, and then reduced to a value comparable to that at 450 rpm. The reduction may be because the higher stirring rate cause minerals to accumulate on the vessel walls. Thus, a rotation rate of 650 rpm was selected as one of the optimal conditions for further research.

The effect of the sulfuric acid (H_2_SO_4_) addition on the extracted zinc was investigated, as shown in Figure 4b. The addition of H_2_SO_4_ slightly improved sphalerite dissolution in the O_3_ + Fe_2_(SO_4_)_3_ system from 10 g/L to 40 g/L. The observed facilitation can be attributed to two reasons. First, the addition of H_2_SO_4_ reduced the hydrolysis of Fe_2_(SO_4_)_3_, and may have decreased the formation of insoluble precipitates. Second, H_2_SO_4_ is capable of dissolving sphalerite directly. However, the addition of 0.5 M H_2_SO_4_ in the presence of 0.3 M Fe_2_(SO_4_)_3_ had little effect on the extracted Zn [32]. In addition, the study showed that as the concentration of H_2_SO_4_ increases, the solubility of O_3_ decreases [19]. Consequently, this promotion is not linear, in the order of 20 > 10 > 40 > 0 g/L H_2_SO_4_ addition. Overall, the addition of H_2_SO_4_ had a weak promoting effect on sphalerite in the O_3_ + Fe_2_(SO_4_)_3_ system. Hence, subsequent experiments set the H_2_SO_4_ addition rate at 10 g/L.

As exhibited in Figure 4c, the effect of temperature (20–45 °C) on sphalerite dissolution was evaluated in the O_3_ + Fe_2_(SO_4_)_3_ system. The values of extracted zinc between 30 and 45 °C were in the order of 40 > 35 ≈ 45 > 30 > 25 ≈ 20 °C. The observed results can be attributed to two overlapping reasons. On the one hand, the reaction constant of sphalerite in the O_3_ + Fe_2_(SO_4_)_3_ system increases with increasing temperature, and on the other hand, the solubility of O_3_ decreases. Likewise, Vinals, Juan, Ruiz, Ferrando, Cruells, Roca, and Casadao [16] leached gold and palladium with aqueous ozone and obtained the fastest leaching rate at approximately 40 °C. In general, temperature had a slightly promoting effect on sphalerite in the O_3_ + Fe_2_(SO_4_)_3_ system within 20 to 45 °C. Subsequent experiments were conducted with the temperature set at 40 °C.

As depicted in Figure 4d, the effect of O_3_ inlet concentration (0–95 mg/L) on sphalerite dissolution was evaluated in the O_3_ + Fe_2_(SO_4_)_3_ system. It is clear that O_3_ can significantly increase the rate and degree of dissolution of sphalerite with Fe_2_(SO_4_)_3_. Indeed, the extracted zinc increased from 17.6% to 74.5% by increasing the O_3_ inlet concentration from 0 to 30 mg/L. Furthermore, the extracted zinc significantly increases with increasing O_3_ inlet concentration, indicating that it plays a crucial role in the dissolution of sphalerite in the O_3_ + Fe_2_(SO_4_)_3_ system. Subsequent experiments were conducted with the O_3_ inlet concentration set at 95 mg/L.

As shown in Figure 4e,f, the effect of Fe_2_(SO_4_)_3_ concentration on sphalerite dissolution was evaluated in the Fe_2_(SO_4_)_3_ and O_3_ + Fe_2_(SO_4_)_3_ systems. The extracted zinc increased linearly with increasing Fe_2_(SO_4_)_3_ concentration in the Fe_2_(SO_4_)_3_ system. The results can be attributed to the fact that the extracted zinc grew as the dose of oxidizer increased [32]. In the O_3_ + Fe_2_(SO_4_)_3_ system, dissolution of sphalerite was significantly facilitated by the addition of Fe_2_(SO_4_)_3_ (0.2–0.8 M). However, the trend of the extracted Zn with increasing Fe_2_(SO_4_)_3_ concentration was opposite to that of the Fe_2_(SO_4_)_3_ system. The results can be attributed to several reasons. The first reason could be that higher concentrations of ferric ions will complex with sulfate to form insoluble sulfates which prevent the reaction from continuing [12,33]. Second, there were multiple reaction pathways in the O_3_ + Fe_2_(SO_4_)_3_ system, and an increase in the Fe_2_(SO_4_)_3_ concentration promoted the slower reaction pathways. The Fe^3+^ in the system acts as both a catalyst and direct reactor, and its direct dissolution of sphalerite is the slower reaction pathway. Moreover, there is extra ozone depletion due to S_8_ from the direct reaction of sphalerite and Fe_2_(SO_4_)_3_. Finally, excessive Fe^2+^ may consume hydroxyl radicals with high redox potential, as Zhang, Dong, and Yang [27] mentioned in Equation (4). Thus, 0.2 M Fe_2_(SO_4_)_3_ was chosen as one of the optimal conditions during sphalerite dissolution in the O_3_ + Fe_2_(SO_4_)_3_ system.

(4) $$Fe^{2+}+\cdot OH\to Fe^{3+}+OH^{-}$$

### 3.3. Properties of the Residue Surface

#### 3.3.1. SEM Images and EDS

**Figure 5:**
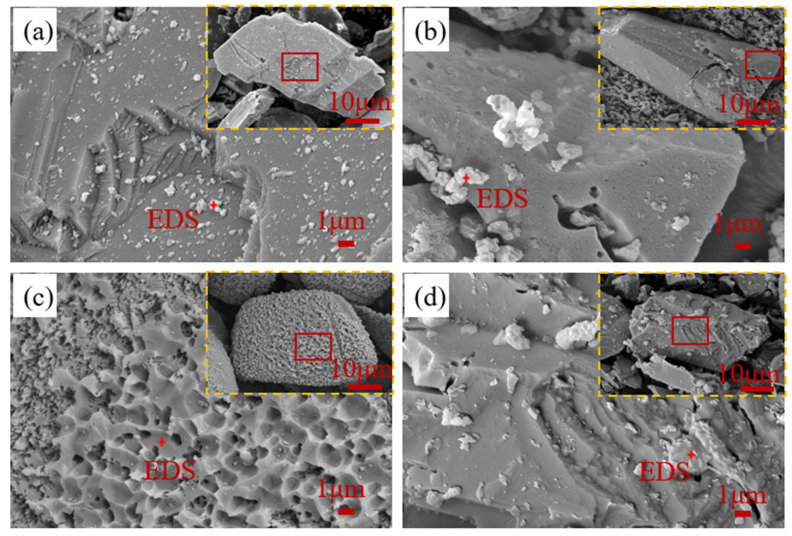
SEM images of sphalerite and dissolved residues from different systems: (**a**) unprocessed; (**b**) O_3_ + Fe_2_(SO_4_)_3_ system; (**c**) O_3_ system; (**d**) Fe_2_(SO_4_)_3_ system; dissolved conditions: [Fe_2_(SO_4_)_3_] = 0.2 M, O_3_ inlet = 95 mg/L and 1.2 L/min, H_2_SO_4_ addition = 10 g/L, T = 40 °C, stirring rate = 650 rpm, reaction time = 2 h.

The surface morphology of the residues after 2 h processed in different systems were observed by SEM (Figure 5a,d). It is easy to obtain the following information. First, Figure 5c,d illustrate that different surface characteristics will form when sphalerite is dissolved by ozone and ferric sulfate. The former are pits with well-defined edges, and the latter are covered by layer products. The unique corrosion surface in Figure 5c may be related to the adsorption site of ozone. Second, the similar surface morphologies of Figure 5b,d indicated that the direct reaction with sphalerite may be mainly Fe_2_(SO_4_)_3_, when ozone is used in combination with Fe_2_(SO_4_)_3_. In addition, many granular products were produced when sphalerite was dissolved in the O_3_ + Fe_2_(SO_4_)_3_ system. However, in the Fe_2_(SO_4_)_3_ system, the product is layered and adsorbed on the sphalerite surface. With the proceeding reaction, the product layer will impede mass transfer, thus causing incomplete leaching of sphalerite. For example, Nikkhou, Xia, and Deditius [12] leached 106–150 μm sphalerite particles using 1.03 M Fe_2_(SO_4_)_3_ in a 1 M citrate solution in the temperature range of 35–130 °C and obtained maximum Zn extractions of 46.1%.

EDS testing further explored the main elemental composition of the residue surface (Table 2). To increase the electrical conductivity of the sample, a layer of platinum was sprayed on the surface. The surface elemental composition of the residues leached by ozone is close to that of unprocessed sphalerite. This may be due to the direct oxidation of sulfur in sphalerite to soluble sulfate by ozone. However, the mass ratio of elemental sulfur increased from 28.28% to 41.53% and 38.22% after leaching in the O_3_ + Fe_2_(SO_4_)_3_ and Fe_2_(SO_4_)_3_ systems, respectively. This result suggests that the product may be elemental sulfur.

#### 3.3.2. Spectral Properties

**Figure 6:**
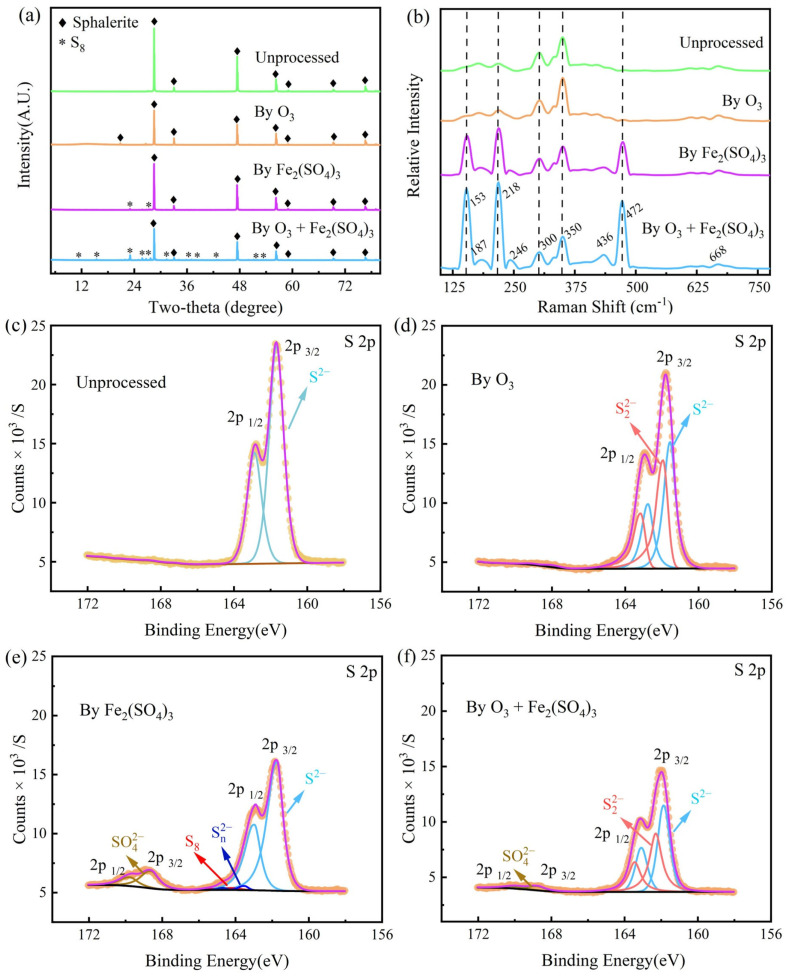
Spectra properties of sphalerite and dissolved residues from different systems: (**a**) XRD spectra; (**b**) Raman spectra; (**c**–**f**) XPS (S 2p). Dissolved conditions: [Fe_2_(SO_4_)_3_] = 0.2 M, O_3_ inlet = 95 mg/L and 1.2 L/min, H_2_SO_4_ addition = 10 g/L, T = 40 °C, stirring rate = 650 rpm, reaction time = 2 h.

The main composition of the surface products of the residues were confirmed by XRD and Raman spectra tests (Figure 6a,b). The primary crystals in the residues are sphalerite, and a small amount of elemental sulfur is produced when Fe_2_(SO_4_)_3_ is present (Figure 6a). In addition, the amount of elemental sulfur produced is significantly more in the O_3_ + Fe_2_(SO_4_)_3_ system than the Fe_2_(SO_4_)_3_ system. The results are mainly because the introduction of ozone plays an essential role in maintaining the concentration of Fe_2_(SO_4_)_3_. The continuous reaction of Fe_2_(SO_4_)_3_ with sphalerite produced large amounts of S_8_. However, as can be seen from the SEM image, this elemental sulfur does not cover the sphalerite surface.

Figure 6b presents the Raman bands of particles on the surface of residues after 2 h of leaching under different oxidation factors. The Raman bands of sphalerite are at 298 (dominant), 309, 340, and 350 cm^−1^, where the band of 350 cm^−1^ is the Zn-S band and the rest are Fe-S bands [34]. However, the band at 350 cm^−1^ in this experiment is the dominant band. This phenomenon can be attributed to the fact that the sphalerite used in this experiment contains only 1.53 wt% Fe. In addition, the study of White [35] indicated that S_8_ had dominant bands at 153, 419, and 472 cm^−1^ and minor bands in 187, 246, and 437 cm^−1^. The positions of these bands are consistent with the last two Raman spectra in Figure 6b. Therefore, it is more certain that the elemental sulfur is generated both in the Fe_2_(SO_4_)_3_ and the O_3_ + Fe_2_(SO_4_)_3_ systems.

The chemical state of elemental sulfur is essential to explore the mechanism of sphalerite dissolution. Hence, an XPS test of elemental sulfur was performed. Figure 6c–f illustrate XPS (S 2p) spectra of the surface of residues after 2 h of leaching under different oxidation factors, and well-fitted results. The 2p orbital usually shows split peaks (2p_1/2_ and 2p_3/2_). Firstly, S^2−^ counts of the samples were significantly reduced in the Fe_2_(SO_4_)_3_ and O_3_ + Fe_2_(SO_4_)_3_ systems. This decrease indicates that a large number of other sulfur products had been produced. Secondly, polysulfides (S_n_^2−^, *n* ≥ 2) were produced on the surface of all residues. This can be attributed to the occurrence of surface relaxation. Because of the unbalanced chemical bond forces of the atoms on new surfaces, S atoms move outward and Zn atoms move inward, forming S-enriched surfaces [35]. Thirdly, while both Raman and XRD spectra showed the presence of S_8_ in the residue surfaces leached in the Fe_2_(SO_4_)_3_ and O_3_ + Fe_2_(SO_4_)_3_ systems. However, S_8_ was only present in Fe_2_(SO_4_)_3_ system in the XPS spectra. This difference may be attributed to the fact that elemental sulfur evaporates in a vacuum for T > 200 K [36]. The S_8_ tested in this XPS may be the residual part after evaporation. Thus, the S_8_ can be identified as one of the reaction products. Finally, the XPS peak for SO_4_^2−^ appears independently because a significant chemical shift occurs in the electron orbitals of S in the presence of four oxygen atoms. The amount of SO_4_^2−^ in the different leaching conditions is in the order of Fe_2_(SO_4_)_3_ system > O_3 +_ Fe_2_(SO_4_)_3_ system > O_3_ system. This phenomenon may be attributed to the fact that the amount of S_8_ covering the residue surfaces is in the order of Fe_2_(SO_4_)_3_ system > O_3 +_ Fe_2_(SO_4_)_3_ system > O_3_ system = 0. Furthermore, SO_4_^2−^ was adsorbed in the porous elemental sulfur. In addition, no S_8_ was present in the solution after complete leaching in the O_3_ and O_3_ + Fe_2_(SO_4_)_3_ systems. This is probably because S_8_ can be oxidized to SO_4_^2−^ by O_3_ [13].

### 3.4. Electrochemical Characterization

**Figure 7:**
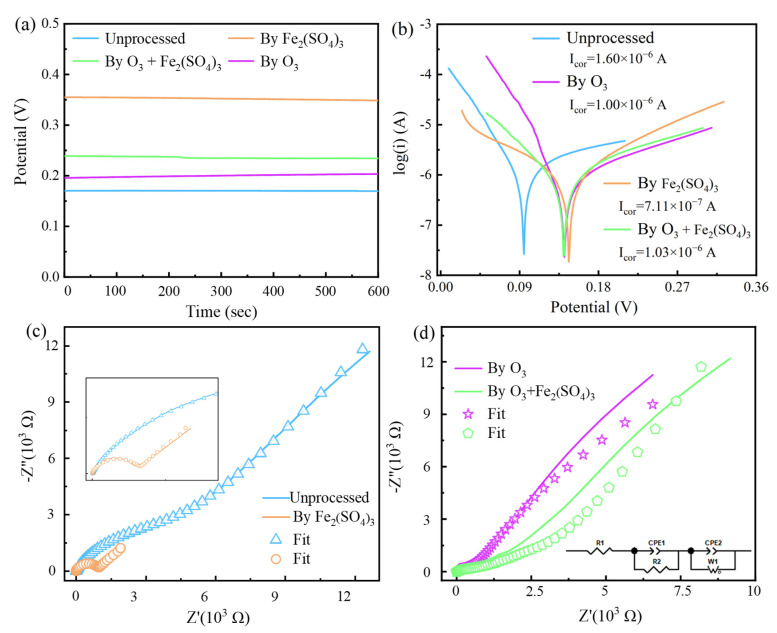
Electrochemical properties of sphalerite carbon paste electrode processed in different systems: (**a**) open circuit potential; (**b**) Tafel curves; (**c**,**d**) Nyquist impedance spectra and equivalent circuit. Processed conditions: [Fe_2_(SO_4_)_3_] = 0.2 M, O_3_ inlet = 95 mg/L and 1.2 L/min, H_2_SO_4_ addition = 10 g/L, T = 40 °C, stirring rate = 650 rpm, reaction time = 2 h.

Loss of electrons is less likely to occur at the electrode with a larger open circuit potential (OCP), i.e., the tendency to be oxidized is smaller [13,37]. Therefore, sphalerite carbon paste electrodes with higher OCPs are more difficult to dissolve by oxidation. Figure 7a illustrates that the most easily oxidized is the unprocessed sphalerite carbon paste electrode. Next is the electrode processed in the O_3_ and O_3_ + Fe_2_(SO_4_)_3_ systems. The OCP of the latter is slightly larger, but much smaller compared to the OCP of the electrode processed in the Fe_2_(SO_4_)_3_ system. This difference in OCP may be due to whether a sulfur layer is produced on the electrode surface.

The corrosion kinetics of minerals can be analyzed by the Tafel test (Figure 7b). The corrosion kinetics are proportional to the corrosion current (I_corr_) [13]. These I_corr_ of electrodes are from larger to smaller unprocessed and processed in the O_3_ + Fe_2_(SO_4_)_3_, O_3_, and Fe_2_(SO_4_)_3_ systems. These results indicate that the corrosion kinetics of the electrode oxidized in the O_3_ + Fe_2_(SO_4_)_3_ system are faster than those in the Fe_2_(SO_4_)_3_ system (Table 3). In addition, the deviation of the corrosion potential (E_corr_) from the OCP may be attributed to the effect of polarization. However, the order of these E_corr_ values in magnitude is consistent with that of OCP. Hence, these corrosion currents are valid for comparing their corrosion kinetics.

The transfer model of charge can be analyzed by electrochemical impedance spectroscopy (EIS). Moreover, Nyquist impedance spectra (Figure 7c,d) and Bode plots were well fitted based on the equivalent circuit (in Figure 7d), and the fitted parameters are shown in Table 4. In the equivalent circuit, R_1_, R_2_, R_3_, and W_1_ represent the solution resistor, surface electric double layer resistance, passivation layer resistance and Warburg impedance, respectively. The resistance and other parameters of the electrodes were relatively similar for different oxidation conditions, except for the electrodes oxidized in the Fe_2_(SO_4_)_3_ system. In particular, the passivation resistance (R_3_) is much higher than that of other electrodes under oxidation conditions. These results indicate that a significant passivation layer was generated at the electrode oxidized in the Fe_2_(SO_4_)_3_ system. And it severely impeded the charge transfer. In contrast, the passivation resistance (R_3_) is less than 10^−5^ at the electrode oxidized in the O_3_ + Fe_2_(SO_4_)_3_ and O_3_ systems. The results indicated that ozone prevents the generation of passivation layers. In addition, the negligible R_3_ of the unprocessed sphalerite electrode is probably due to polishing. Therefore, good charge transfer efficiency can be provided when sphalerite is oxidized with ozone and iron(III) sulfate.

### 3.5. Dissolution Kinetics

**Figure 8:**
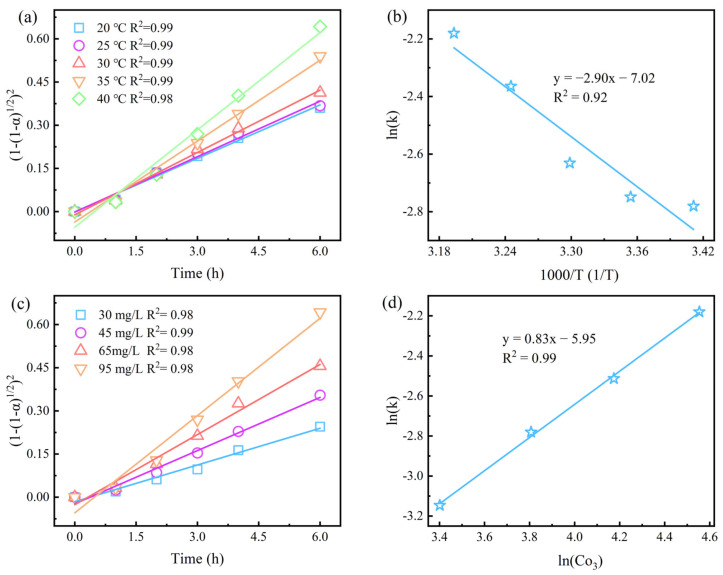
Kinetic fitting of sphalerite dissolving in Fe_2_(SO_4_)_3_-O_3_ system: (**a**,**b**) kinetic fit and graph of ln(k)–1/T in 20–40 °C; (**c**,**d**) kinetic fit and plot of ln(k)–ln[O_3_] in 30–95 mg/L O_3_ inlet. Experimental conditions: [Fe_2_(SO_4_)_3_] = 0.4 M, O_3_ inlet = 95 mg/L and 1.2 L/min, H_2_SO_4_ addition = 10 g/L, T = 40 °C, stirring rate = 650 rpm, reaction time = 2 h.

The dissolution of sphalerite in the Fe_2_(SO_4_)_3_-O_3_ system is intrinsically a liquid–solid reaction. Furthermore, the reaction rate was significantly affected by O_3_ concentration and stirring rate, and slightly by temperature. Thus, the rate-controlling step of the reaction may be the diffusion process of O_3_. To verify this speculation, the Jander (cylindrical diffusion) equation (Equation (5)) was used to fit the leaching data at different temperatures and different O_3_ concentrations [38]. As seen in Figure 8a,c, the good fit of this equation to the leaching data suggests that it can be used to explain the kinetics of sphalerite dissolution in the Fe_2_(SO_4_)_3_-O_3_ system. The apparent activation energy of the reaction process was calculated to be 24.11 kJ/mol based on the Arrhenius equation, since ln(k) versus 1/T is excellently linear in the range of 20–40 °C, as seen in Figure 8b. The values of activation energy indicate that the dissolution reaction of sphalerite in the Fe_2_(SO_4_)_3_-O_3_ system is mainly controlled by diffusion.

(5) $${\left(1-{\left(1-\alpha \right)}^{1/2}\right)}^{2}=kt$$

To further understand the effect of O_3_ concentration on sphalerite dissolution in the Fe_2_(SO_4_)_3_-O_3_ system, the plot of ln(k) versus ln[O_3_] was fitted. As exhibited in Figure 8c an excellent linear relationship was observed between them, confirming the linear effect of O_3_ concentration on the extracted zinc. In addition, the slope value (0.83) represents the empirical order of sphalerite dissolution regarding the O_3_ inlet concentration in the Fe_2_(SO_4_)_3_-O_3_ system (Figure 8d). Furthermore, it can be seen from the SEM images that there is no product layer in the presence of ozone, indicating that it is not product layer diffusion. Therefore, it is more certain that the dissolution rate of sphalerite in the Fe_2_(SO_4_)_3_-O_3_ system is mainly controlled by ozone diffusion processes.

### 3.6. Possible Reaction Mechanisms

**Figure 9:**
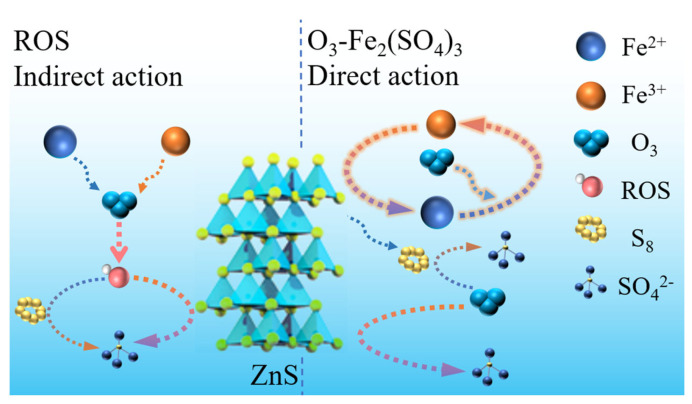
Mechanism model for dissolution of sphalerite in the Fe_2_(SO_4_)_3_-O_3_ system.

According to the above surface properties and experimental results, a possible mechanism for sphalerite dissolution in the Fe_2_(SO_4_)_3_-O_3_ system has been proposed, as shown in Figure 9. (1) Direct oxidation of O_3_ and Fe_2_(SO_4_)_3_ with sphalerite. Fe_2_(SO_4_)_3_ can react directly with sphalerite and the characteristic product is S_8_. The reaction of O_3_ with sphalerite produces little or no elemental sulfur, and the products are likely to be soluble sulfur oxides (SO_4_^2−^). Furthermore, O_3_ oxidizes the Fe^2+^ produced by sphalerite reduction to Fe^3+^ and S_8_ to soluble sulfur oxides. (2) ROS (·OH, ^1^O_2_ and ·O_2_^−^) generated by Fe(II)- and Fe(III)-catalyzed O_3_ oxidizing sphalerite. These ROS act similarly to O_3_, but have a more powerful oxidizing capacity than it. (3) The dissolution reaction of sphalerite in the Fe_2_(SO_4_)_3_-O_3_ system is mainly controlled by diffusion, and the rate of sphalerite dissolution depends mainly on the concentration of dissolved O_3_.

The specific process of ROS generation is shown in Equations (6)–(12) [26,30]. The generation of ·OH from Fe^2+^ and Fe^3+^ with O_3_ is essentially an acid-consuming process, and thus they can be used as catalysts under acidic conditions. Moreover, there may be two main reasons why ·O_2_^−^ among the ROS played a dominant role in sphalerite dissolution in the Fe_2_(SO_4_)_3_-O_3_ system. First, Fe^2+^ and Fe^3+^ readily form surface hydroxyl groups with water, which react with ozone to produce large quantities of ·O_2_^−^ and ·OH_2_. Second, ·OH_2_ is capable of producing ·O_2_^−^ and ·OH in different pathway. More ·OH_2_ may be converted to ·O_2_^−^ during sphalerite dissolution in the Fe_2_(SO_4_)_3_-O_3_ system.

(6) $$Fe^{2+}+O_{3}\to Fe^{3+}+\cdot O_{3}^{-}$$

(7) $$O_{3}^{-}+H^{+}\to O_{2}+\cdot OH$$

(8) $$Fe^{3+}+O_{3}+H_{2}O\to FeO^{2+}+H^{+}+\cdot OH+O_{2}$$

(9) $$O_{3}+OH^{-}\to \cdot O_{2}^{-}+\cdot HO_{2}$$

(10) $$HO_{2}+O_{3}\to 2O_{2}+\cdot OH$$

(11) $$HO_{2}\to \cdot O_{2}^{-}+H^{+}$$

(12) $$OH+\cdot O_{2}^{-}\to^{1}O_{2}+OH^{-}$$

## 4. Conclusions

The proposed O_3_-Fe_2_(SO_4_)_3_ leaching process is an excellent alternative for the efficient and eco-friendly recovery of valuable metals from sphalerite at atmospheric pressure. Under the optimum conditions ([Fe_2_(SO_4_)_3_] = 0.2 M, O_3_ inlet = 95 mg/L and 1.2 L/min, H_2_SO_4_ addition = 10 g/L, T = 40 °C, stirring rate = 650 rpm), the extracted zinc achieved 97.8% and was significantly higher than systems O_3_ (82.1%) and Fe_2_(SO_4_)_3_ (16.4%). At the same time, the electrochemical properties of ozonated sphalerite are similar to those of sphalerite, which indicates that ozone prevents the generation of passivation layers.

ROS (·OH, ^1^O_2_ and ·O_2_^−^) from O_3_ decomposition played a significant role in the dissolution of sphalerite, especially ·O_2_^−^. Furthermore, the sulfur products of sphalerite in the Fe_2_(SO_4_)_3_ and O_3_ systems are S_8_ and sulfates, respectively. S_8_ was also produced in the O_3_-Fe_2_(SO_4_)_3_ system, but without inhibiting the dissolution of sphalerite. Moreover, the apparent activation energy was 24.11 kJ/mol based on kinetic fitting, which indicated that the controlling step of the reaction was mainly a diffusion process. In addition, the empirical order (0.83) of sphalerite dissolution regarding the O_3_ inlet concentration was obtained. These kinetic results revealed that the O_3_ concentration in the solution is the critical factor in determining the rate of sphalerite dissolution in the O_3_-Fe_2_(SO_4_)_3_ system.

## Figures and Tables

**Table 1:** The water quality parameters of distilled water.

Aqueous Solution	pH	Total Dissolved Solids (mg/L)	Electrical Conductivity (μS/cm)	Total Hardness
distilled water-1	7.15	0.44	0.072	NA
distilled water-2	7.07	0.29	0.065	NA

**Table 2:** Elemental composition of the residue surface.

Parameters	Zn (wt%)	S (wt%)
a	66.34	28.28
b	50.60	41.53
c	67.52	27.89
d	58.61	38.22

**Table 3:** Parameter values of the Tafel test curves.

Parameters	Cat Slp (1/v)	Ano Slp (1/v)	Lin Pol R (ohms)	Corr I (A)
Unprocessed	25.0	4.0	534,38	1.600 × 10^−6^
By O_3_	25.0	6.5	146,393	1.000 × 10^−6^
By Fe_2_(SO_4_)_3_	10.5	10.0	194,038	7.112 × 10^−7^
By O_3_ + Fe_2_(SO_4_)_3_	15.5	6.0	127,008	1.033 × 10^−6^

**Table 4:** Impedance parameter values of the equivalent circuit.

Parameters	R_1_	CPE-T_1_	CPE-P_1_	R_2_	CPE-T_2_	CPE-P_2_	R_3_	W_1_-R	W_1_-T	W_1_-P
Unprocessed	7.24	6.84 × 10^−7^	1.118	2.59	4.93 × 10^−5^	0.778	0.002	8432	5.94	0.297
By Fe_2_(SO_4_)_3_	6.70	3.98 × 10^−6^	1.211	63.58	1.62 × 10^−5^	1.042	733	8996	456.21	0.496
By O_3_	6.06	1.03 × 10^−6^	1.516	8.60	3.72 × 10^−7^	1.464	<10^−5^	5418	5.88	0.477
By O_3_ + Fe_2_(SO_4_)_3_	6.09	2.04 × 10^−7^	1.476	26.55	3.93 × 10^−7^	1.357	<10^−5^	7600	6.33	0.390

## Data Availability

Some or all data that support the findings of this study are available from the corresponding author upon reasonable request.
